# British Medicinal Plants

**Published:** 1890-09

**Authors:** Alfred Heath

**Affiliations:** London, England


### BRITISH MEDICINAL PLANTS.

ALFRED HEATH, M. D., F. L. S., LONDON, ENGLAND.

### RANUNCULACEÆ (CONTINUED).

*Aconitum Napellus* (Monkshood, Wolfsbane).—In England this plant is rare in a *wild* state, and is generally believed to be introduced. It is now completely naturalized and included in works on English botany; as a garden plant, it is one of the commonest, and its tall, showy spikes of dark-blue flowers, surmounting the dark, deeply-cut leaves, are worthy of a place anywhere.

This famous remedy, appropriately named "the homœopathic lancet," on account of its power in reducing fever without taking blood, as in the old school (thanks to Homœopathy, this pernicious practice is seldom heard of now), has probably done more in saving life than any other drug, as tens of thousands can testify: it stands, *par excellence*, at the head of the long list of drugs introduced to the world through the genius of Hahnemann.

Its active principle, *Aconitine*, or now more generally called *Aconitia*, is one of, if not the most deathly preparation known to man; a very small portion of it causes death, and there is no known antidote. It seems to me that the more deathly a drug is in its action as a poison, the greater are its virtues, and the more powerful is it in the saving of life, when given according to the homœopathic "law of cure." Instance such invaluable medicines as *Aconitum*, *Arsenicum*, *Belladonna*, *Conium*, *Digitalis*, *Nux-vomica*, *Phosphorus*, *Veratrum*, *etc.*

Since the report of a celebrated case of poisoning by *Aconitia*, a large number of people have been very much afraid of using any preparation called by the name of Aconite, and it should be explained that, in Homœopathy, we do not officially use Aconitia at all, being far too dangerous and also not in accordance with the teaching of the system which advocates the use of every part of a plant, or of some special part, and not the poisonous or active part principle only, unless that alone has been proved. The preparation used in Homœopathy is a tincture made from the whole plant, or from the root only, and in either case contains but a small proportion of Aconitia. This $\phi$, or matrix tincture, is seldom given, but only dilutions made from it, of various strengths, such as 1 dec., 2 dec., 3 dec., and so on, so that one may give the tenth, one-hundredth, or one-thousandth of a drop or less, and, strange as it may appear to those unacquainted with the system, the smaller dose will generally act better than the larger one, as it must not be forgotten that *the medicine, to be homœopathic, must be capable of producing in a healthy body a fac simile of the symptoms produced by the disease. Aconite*, in small doses, is a most potent remedy against the *dry heat, flushed face*, and *restlessness of fever*. Any one, with a few drops of this medicine, can *prove* the truth of the homœopathic law. Let him or her, being a good and sound sleeper, take, on going to bed, a drop or two of the tincture of *Aconite*. The result will be a more or less sleepless night, *restless*, and *tossing about*, heat, etc. ; on the other hand, let them be sleeping badly from *other* causes, with this *restlessness*, heat, and tossing about, a drop or two of the same tincture, in almost any dilution, will remove all these sensations, and the person will sleep soundly from *natural* causes, the thing that prevented healthy sleep having been removed in accordance with the homœopathic law of similars. This is precisely the action of *every* medicine, but before a medicine can be used homœopathically, its symptoms must be known. It *must* be proved on the healthy to know its symptoms or sphere of action.

*Aconite* has, perhaps, made more converts to Homœopathy than any other drug, because its effect, in fever, is so marked. A case came under my care, some time ago, of a child suffering from congestion, bordering on inflammation, of the lung, with *incessant*, dry cough, *great restlessness, flushed face, high temperature, skin dry, burning ;* it had had *no* sleep for a day and a night, and could not be laid down for a moment ; one dose of Aconite allayed the cough and fever, and within five minutes the child was lying asleep in its cot, and slept for some hours during the night. The fever was subdued, and it eventually made a good recovery. After such facts as these, which come under the notice of thousands, is it a wonder that Homœopathy increases? The only wonder is that it does not increase faster. Hundreds of cases, supposed to be cured by the allopathic doctor, are, in reality, cured by a few doses of homœopathic medicine, given by the parents, and often after the doctor has pronounced the case to be hopeless. Very little notice was taken of this drug as a medicine, until after its introduction by Hahnemann, and, until recently, it was only used as an external remedy in various kinds of neuralgic and rheumatic pains. Many cases are related of its employment, allopathically, in chronic rheumatism, where this disease, though of years' duration, and having withstood the use of other powerful medicines, such as Mercury, Opium, Antimony, Conium, etc., was, in a short time, cured by Aconite. The reason of this is plain. Aconite on the *healthy*, produces, amongst other things, "pains as from a bruise, weakness and swelling of the arms and shoulders, heaviness, numbness of the fingers, paralytic weakness of the arm and hand, a sensation of drawing in the arms, deadness of hands, hot hands and cold feet, tingling in the fingers, similar pains in the legs, pains which force one to cry out at every step, want of strength and of stability in the joints of the hip and of the knee, stiffness of the legs on moving, pains in the insteps, with despair and fear of death, numbness of the legs, heaviness of the feet, pains, as if bruised, in the neck, back, and loins, and painful stiffness in the nape of the neck. It also produced dry, burning skin, yellowish color of the skin, spots similar to flea-bites on the hands and body (it is one of the first remedies in measles, purpura, etc.), sleeplessness from anxiety, with constant agitation and tossing, starting in sleep, anxious dreams, nightmare, dreams with a sort of clairvoyance (this last symptom seems peculiar to the Ranunculaceæ), dry, burning heat, with extreme thirst, sometimes preceded by shiverings and tremblings; heat chiefly in the head and face, with redness of the cheeks, shuddering over the entire body; shivering, if uncovered in the least, while the heat exists, great agitation and tossing of the body; with anguish, inconsolable irritability, cries, tears, groans, complaints and reproaches, fearful anticipations of approaching death, and a strong disposition to be angry, to be frightened, and to quarrel, the least noise, even music, appears insupportable; delirium," etc. For the remainder of the mental symptoms, and also for its effects on other parts of the body, see the proving in Jahr's *Materia Medica.*

Unfortunately a great deal of the Aconite in the market is not the true Aconitum Napellus, from which the proving was made, and which is the only one that should be used. There are a large number of Aconites found wild in Europe, with totally different medicinal principles, and with different-shaped and colored flowers, and flowering at different times, and as most of the Aconite used is the imported root, and as many of the roots are alike in general appearance, and are collected by persons totally ignorant of botany or of the botanical character of Aconitum Napellus, we have no guarantee that we have the true variety. Consequently, unless we can see the flower and know also the time of flowering (the A. Napellus flowers about May or June), the only way to obtain the genuine variety is to grow it.

